# Genotype by environment interaction for growth and *Dothistroma* resistance and clonal connectivity between environments in radiata pine in New Zealand and Australia

**DOI:** 10.1371/journal.pone.0205402

**Published:** 2018-10-12

**Authors:** Yongjun Li, Heidi S. Dungey, Mike Carson, Sue Carson

**Affiliations:** 1 Scion (New Zealand Forest Research Institute), Rotorua, New Zealand; 2 Agriculture Victoria, AgriBio Centre, Bundoora, Victoria, Australia; 3 Forest Genetics Ltd., Ngongotaha, Rotorua, New Zealand; 4 Carson Associates Ltd, Ngongotaha, Rotorua, New Zealand; Natural Resources Canada, CANADA

## Abstract

Twenty-eight clonal trials of radiata pine planted across Australia and New Zealand were used to investigate genetic variation and genotype by environment (G×E) interaction for diameter-at-breast-height (DBH), height and *Dothistroma* resistance (DO_R). The average narrow-sense heritabilities were 0.11, 0.21 and 0.30 while the average broad-sense heritabilities were 0.27, 0.34 and 0.40 for DBH, height and *Dothistroma* resistance, respectively. *Dothistroma* resistance was assessed as the percentage of needles that were not affected by Dothistroma needle blight. G×E interactions were analysed using an approximate reduced factor analytic model. Apparent G×E interactions were estimated for DBH, height and *Dothistroma* resistance. Estimates of G×E interactions and their standard errors were strongly influenced by the level of connectivity between trials, in terms of common clones and common parents. When there was sufficient connectivity between trials (more than 30% common clones between trials), a high level of G×E interaction was found for DBH and height but not for *Dothistroma* resistance. In two simulated clonal trials planted in two environments, low connectivity between environments resulted in a lower estimated genetic correlation between environments with an increased standard error. These results suggest that the number of clones in common between clonal trials is a key factor for inclusion in future experimental designs for estimating G×E interaction. When designing clonal trials for use in multiple environments for accurately estimating the level of G×E, if the resource for creating connectivity between environments is limited, at least 30% of the clones need to be in common between environments.

## Introduction

Radiata pine (*Pinus radiata*) is a fast-growing pine species, native to the Central Coast of California and both Guadalupe Island and Cedros Island of Mexico [1]. Its timber is used for paper making, reconstituted board products and plywood manufacture, house construction and furniture making [2]. There are over four million ha of planted radiata pine worldwide, with New Zealand (1.72 million ha) and Australia (0.77 million ha) among the countries with plantations [1]. Radiata pine plantations in Australia are in the southern part of the country, distributed in New South Wales, Victoria, South Australia, Tasmania and Western Australia. In New Zealand, the species is planted across the country, with 70% in the North Island and 30% in the South Island [3]. The New Zealand forest industry has a vision to significantly improve forest profitability by doubling productivity on a per hectare basis by 2025 while also improving wood quality and increasing resistance to pests and diseases [4]. Current breeding stock will need to be improved genetically in order to achieve this increased productivity target.

Forest tree breeding programmes have played an important role in improving forest productivity around the world. The radiata pine breeding programmes in New Zealand and Australia began in the 1950s and initially focused on growth, form and health traits, later extending to wood quality traits [5–7]. Radiata pine has been bred for three generations in Australia since the 1950s, with realised genetic gain up to 33% for volume from the first generation and more than 10% gain predicted from the second generation [8]. A growth and form (GF) rating system based on the levels of genetic gain in seedlots or planting stock has been used to evaluate the potential of improved genetic material in New Zealand [7, 9, 10]. Compared with an unimproved seedlot, the realized genetic gain for volume in New Zealand increased 12% for an early seedlot rated GF12 and 19% for a mid-1980s standard controlled cross rated GF22 [11]. In 1998, the GF rating was replaced by a new GF Plus rating system developed by the Radiata Pine Breeding Company (www.rpbc.co.nz). Compared with unimproved seedlots, a GFPlus 25 seedlot had 25% increase in realized genetic gain for total standing volume at age 30 years, and each unit increase in GF Plus rating was associated with a 1.51% increase in volume growth rate [12].

Growth and *Dothistroma* resistance are important selection traits for radiata pine in New Zealand and Australia [6]. Dothistroma needle blight, caused by *Dothistroma septosporum* (Dorog.) M. Morelet [13], is a serious needle disease affecting radiata pine in New Zealand and Australia [14–16]. It occurs in young trees up to age 15, affecting the current year’s foliage. Infection starts from the base of the tree progressing upwards, with defoliation most apparent in spring. The typical symptom is distinct brick-red bands (1–3 mm wide) around the needles that can appear within weeks of infection and can sometimes still be seen after the needles have died [17]. This disease can cause growth loss due to severe defoliation of radiata pine and this is its most significant econmic impact [14]. On an individual tree basis, reduction in volume increment is directly proportional to the average disease level over a period of at least 3 years. In New Zealand, the disease has resulted in growth loss of up to 70%, particularly in moist warm climates [18].

In order to produce improved forest tree germplasm, genotypes are usually tested across a wide range of environments. Genotype by environment interactions (G×E) have been observed in a number of forest tree species and can be problematic for breeding programmes [19,20]. G×E is defined as where the performance of a genotype that is superior in one environment might be inferior in another environment [21,22]. G×E as estimated in statistical analyses can be an expression of either differences in the size of the genetic variance across trials, and/or changes in ranking of genotypes. Changes in genotype ranking are considered more important in terms of deployment of genetically-improved trees. High levels of G×E have been reported for growth traits in radiata pine [21–27]. Low levels of G×E have been reported in most studies for stem straightness [28,29], branch angle [30], branch size[31], branch habit [32] and malformation [32]. No significant G×E has been found for wood basic density [33–35], predicted modulus of elasticity [33] and resistance to damage caused by the *Dothistroma* pathogen [28].

The ability to realise expected genetic gains in the forest depends on the accuracy of estimated breeding values of selection candidates for the deployed population. G×E complicates the design and interpretation of trials across environments that are used for estimation of breeding values of selected genotypes intended for deployment, whether as seed orchard progeny, or tested clones. It reduces the profitability of the forest venture to the extent that additional investment in a more ‘fine-grained’ series of screening trials may be justified. Breeders interested in optimising gains in deployed tree stocks often address G×E either by selecting for stable genotypes that are not sensitive to environmental changes, or selecting genotypes for specific environments in order to maximise genetic gain on specific sites, or site types [36]. In New Zealand G×E for growth rate was considered not sufficiently important to require the development of regionalised radiata pine breeding programmes [28]. However, it was recognised that there is sufficient G×E for growth rate to provide forest growers with the opportunity to increase genetic gain in their forests by selecting genotypes that are best suited to each individual environment, provided that they are willing to invest in the additional costs of trialling that this would require [26,37,38].

Genetic correlation between environments has been used as a measure of G×E in plants and livestock. The accurate estimation of genetic correlation between environments depends on the connectivity or relationship shared between individuals planted in two or more environments. As a first objective, this study used clonal trials planted in New Zealand and Australia to estimate the level of G×E for growth and *Dothistroma* resistance in radiata pine. The connectivity between these clonal trials was realized through sharing common parents or clones between trials at different levels. The secoand objective of this study was to investigate the relationship between the level of G×E and the connectivity between trials. Further the clonal testing used in this study allowed us to examine the effectiveness of these types of trials for estimation of G×E.

## Materials and methods

### Genetic material and phenotypes

Nine single-tree-plot clonal trials (S01-S09) used in this study were from planted forest estates across southern Australia and 19 single-tree-plot clonal trials (S10-S28) were from planted forest estates across New Zealand (Table 1). Three traits were analysed: diameter-at-breast-height (DBH), height and *Dothistroma* resistance (DO_R). DBH was assessed in centimeters at breast height (1.3 metres above the ground in Australia and 1.4 metres above the ground in New Zealand). Total tree height was assessed in metres. Infection by Dothistroma needle blight on individual trees was assessed as the percentage of the total needles present on the tree that was diseased [39]. Scoring was undertaken in 5% increments, i.e., 5, 10, 15, 20, 25, etc. A score of 40% indicated that 40% of the needles present were diseased. DO_R was calculated as the percentage of needles that were not affected by Dothistroma needle blight, which was equal to 100% minus the percentage of needles that were affected by the disease. Ages at assessment are expressed in months from planting. In total, there were 29,381 DBH observations in 21 trials, 29,911 height observations in 24 trials and 6,608 Dothistroma needle blight damage observations in five trials. Observations used in this analysis were from a single assessment in every trial. Progeny of seedlots used as controls were removed from this analysis and were not included in Table 1.

**Table 1:** Establishment year, location (Longitude East and Latitude South) of trials, experimental design features and descriptive summary of phenotypes of DBH, height and *Dothistroma* resistance of the trials used in this study. S01-S09 are located in Australia and S10-S28 are located in New Zealand.

Trial	Year	Longitude East	Latitude South	No of clones	No of full-sibs	No of mothers	No of Fathers	DBH (cm)				Height (m)				*Dothistroma* resistance (%)			
								Age^ξ^	N	Mean	SD	Age	N	Mean	SD	Age	N	Mean	SD
S01	2003	148°01'45"	-35°32'46"	245	0	15	16	79	1068	12.78	2.52	79	1068	8.22	1.34	-	-	-	-
S02	2003	149°34'38"	-33°25'09"	302	0	15	16	55	2250	8.81	2.31	55	2254	4.97	1.02	-	-	-	-
S03	2003	149°14'32"	-36°54'41"	357	0	15	16	55	3011	7.475	2.09	55	3027	4.44	1.00	-	-	-	-
S04	2003	140°35'59"	-37°40'59"	305	0	14	15	47	2141	7.75	1.81	47	2144	4.79	0.88	-	-	-	-
S05	2003	146°45'09"	-36°24'21"	313	0	15	16	59	2558	8.42	2.14	59	2559	5.57	1.22	-	-	-	-
S06	2003	140°58'09"	-37°49'03"	195	0	14	15	48	1662	9.47	2.15	48	1659	5.03	0.81	-	-	-	-
S07	2003	115°38'22"	-33°19'32"	212	0	13	14	74	2379	11.50	2.43	-	-	-	-	-	-	-	-
S08	2004	149°03'01"	-35°12'09"	149	0	10	10	61	1133	11.76	2.12	61	1134	7.27	0.88	-	-	-	-
S09	2005	146°12'44"	-38°24'53"	195	0	11	11	-	-	-	-	44	1423	4.18	0.69	-	-	-	-
S10	1999	178°19'10"	-37°53'34"	169	0	11	11		-	-	-	-	-	-	-	43	1396	82.75	13.06
S11	1999	176°44'29"	-39°46'16"	129	0	11	11	99	590	24.02	3.09	99	590	14.92	1.60	-	-	-	-
S12	2000	174°46'34"	-41°17'11"	335	0	13	11	57	923	10.07	2.79	47	927	3.60	0.77	-	-	-	-
S13	2000	177°49'59"	-38°150'0"	335	0	15	13	-	-	-	-	47	1040	3.78	0.78	-	-	-	-
S14	2000	176°44'29"	-39°46'16"	168	0	11	8	88	712	19.70	3.19	88	708	12.19	1.76	54	844	87.35	7.90
S15	2000	168°14'18"	-45°58'12"	168	0	11	8	46	663	2.80	0.79	46	725	2.36	0.38	-	-	-	-
S16	2000	173°17'02"	-41°16'14"	337	0	14	13	-	-	-	-	33	1181	2.42	0.45	-	-	-	-
S17	2000	169°38'04"	-44°49'40"	336	0	15	13	-	-	-	-	33	1141	1.95	0.44	-	-	-	-
S18	2000	173°25'18"	-41°34'21"	167	0	15	13	28	636	1.67	0.79	28	761	1.87	0.43	-	-	-	-
S19	2001	176°49'51"	-38°03'50"	127	0	11	13	-	-	-	-	35	743	4.95	0.77	43	830	62.16	13.26
S20	2001	176°37'46"	-38°10'39"	126	0	10	12	65	813	11.90	2.46	35	840	2.46	0.57	65	891	70.70	16.01
S21	2001	176°44'29"	-39°46'16"	255	0	11	15	75	1729	19.78	2.37	75	1726	12.50	1.43	-	-	-	-
S22	2001	178°04'24"	-37°49'37"	211	0	15	16	70	893	17.65	3.52	70	892	8.42	1.28	-	-	-	-
S23	2001	174°46'34"	-41°17'11"	168	0	13	13	68	948	13.09	2.93	37	975	2.23	0.59	-	-	-	-
S24	2001	168°31'45"	-46°08'21"	169	0	12	13	60	965	7.62	1.91	-	-	-	-	-	-	-	-
S25	2003	176°33'52"	-38°24'30"	441	197	17	20	46	2716	7.18	1.47	-	-	-	-	28	3174	61.21	22.15
S26	2005	176°33'52"	-38°24'30"	54	444	10	15	55	1254	9.75	1.59	28	1314	1.73	0.34	-	-	-	-
S27	2005	176°33'52"	-38°24'30"	54	438	10	15	-	-	-	-	28	1252	1.26	0.33	-	-	-	-
S28	2005	176°33'52"	-38°24'30"	54	443	10	15	54	1220	10.15	1.69	26	1291	1.65	0.27	-	-	-	-

^ξ^ Unit of age is month.

### Statistical analysis

Heritabilities and genetic correlations between trials were analysed using an individual-tree linear mixed model, implemented in ASReml-R [40]:

$$y=Xb+Z_{a}u+Z_{d}d+e$$ (1)

where ***y*** is a vector of trait phenotypic observations, ***b*** is a vector of fixed effects (including overall mean and trial mean), ***u*** is the vector of random additive genetic effects, ***d*** is a vector of random non-additive genetic effects, and e is a vector of random residual effects. ***X***, ***Z***_***a***_, and ***Z***_***d***_ are known incidence matrices relating the observations to effects of *b*, *u*, and ***c***, respectively. Experimental features of replicates, sets within replicates, row, block, and plot were fitted as random effects.

The level of G×E was measured by genetic correlation between environments. The higher the genetic correlation between environments the lower the level of G×E between environments. The genetic correlation matrix among all trials was estimated using the approximate reduced animal model of the factor analytic model [26] with an order of 2 (denoted as FA2). In the linear mixed model (1), the random additive genetic effects ***u*** have a normal distribution with ***u***~*N*(0,***G*_*A*_**⊗***A***), where $G_{A}=\left[\begin{array}{ccc} \sigma_{a_{1}}^{2} & \cdots & \sigma_{a_{1}a_{t}}\\ \vdots & \ddots & \vdots \\ \sigma_{a_{t}a_{1}} & \cdots & \sigma_{a_{t}}^{2} \end{array}\right]$, where $\sigma_{a_{i}}^{2}$ is the additive genetic variance for trial *i*, $\sigma_{a_{i}a_{j}}$ is the additive genetic covariance between trial *i* and trial *j*, ***A*** is the numerator relationship matrix, *t* is the number of trials, ⊗ denotes the Kronecker product; the non-additive genetic effects *d* have a normal distribution with ***d***~*N*(0,***G*_*d*_**⊗***I***), where $G_{d}=\left[\begin{array}{ccc} \sigma_{d_{1}}^{2} & \cdots & 0\\ \vdots & \ddots & \vdots \\ 0 & \cdots & \sigma_{d_{t}}^{2} \end{array}\right],\;\sigma_{d_{i}}^{2}$ is the non-additive genetic variance for trial *i* and ***I*** is the identity matrix; and the residual effects ***e*** have a normal distribution with ***e***~*N*(0,*R*⊗***I***), where $R=\left[\begin{array}{ccc} \sigma_{e_{1}}^{2} & \cdots & 0\\ \vdots & \ddots & \vdots \\ 0 & \cdots & \sigma_{e_{t}}^{2} \end{array}\right]$, where $\sigma_{e_{i}}^{2}$ is the residual variance for trial *i*. The FA2 model for the additive genetic effects of *m* genotypes in *t* trials can be modelled as ***u* = (Λ⨂I_m_)f+*δ*** [26,41], where Λ is the *t* **× 2** matrix of loadings, **I**_**m**_ is an identity matrix of dimension *m* **×** *m*, **f** is the 2*m* × 1 vector of scores and ***δ*** is the *mt* × 1 vector of genetic regression residuals. Var(***u***) = (**ΛΛ′+*Ψ*)⨂I_m_** with the assumption of var(f) = I_**2m**_, var(***δ***) = ***ψ*⨂I_m_**, where ***ψ*** is a *t* ***×*** *t* diagonal matrix with a variance (called a specific variance) for each environment, and the vectors of random effects **f** and ***δ*** are mutually independent as a multivariate Gaussian distribution with zero means. The between environment genetic variance matrix is defined as ***G***_***a***_ = (**ΛΛ**′+***Ψ)*. *G***_***a***_ can be estimated with the REML algorithms as ${\hat{G}}_{a}=(\hat{\Lambda }{\hat{\Lambda }}^{\prime }+\hat{\psi }]$ and can be converted to the genetic correlation matrix between trials ${\hat{C}}_{a}={\hat{D}}_{a}{\hat{G}}_{a}{\hat{D}}_{a}$, where ${\hat{D}}_{a}$ is a diagonal matrix with elements given by the inverse of the square roots of the diagonal elements of ${\hat{G}}_{a}$. The standard error of estimated genetic correlations between trials were extracted from ASReml-R output. Heritabilities for DBH, height and *Dothistroma* resistance were estimated using the mixed linear model (1) with heterogeneous genetic variances across trials for the additive genetic effects, the non-additive genetic effects and residual effects. The narrow-sense heritability for trial *i* ($h_{i}^{2}$) was estimated as $h_{i}^{2}=\frac{\sigma_{a,i}^{2}}{\sigma_{a,i}^{2}+\sigma_{d,i}^{2}+\sigma_{e,i}^{2}}$ and the broad-sense heritability for trial *i* ($H_{i}^{2}$) was estimated as $H_{i}^{2}=\frac{\sigma_{a,i}^{2}+\sigma_{d,i}^{2}}{\sigma_{a,i}^{2}+\sigma_{d,i}^{2}+\sigma_{e,i}^{2}}$, where $\sigma_{a,i}^{2}$ is the ***i***th diagonal element of *G*_*a*_ for trial *i*, $\sigma_{d,i}^{2}$ is the ***i***th diagonal element of *G*_*d*_ for trial *i*, $\sigma_{e,i}^{2}$ is the ith diagonal element of *R* for trial *i*, and $\sigma_{a,i}^{2}+\sigma_{d,i}^{2}+\sigma_{e,i}^{2}$ is the total phenotypic variance for trial *i*.

### Simulation

A simulation was conducted to mimic the sizes of trials analysed (Table 1) with similar numbers of parents, families and clones across two environments. The aim of this simulation was to investigate the effect of connectivity between environments on the estimation of genetic correlation between environments and its standard error. The simulation started with a base population of 3,000 unselected trees. Twenty-five males and twenty-five females were selected and each had two random crosses to form 50 control-pollinated families. Ten clones were generated from each family and 10 ramets generated from each clone, with 500 clones and 5,000 plants generated in total. In order to test the importance of the type of connectivity, we simulated trials that were connected through both common families and common clones between environments. We simulated 10 levels of families in common: 5 (Ten percent of families in common), 10, 15, 20, 25, 30, 35, 40, 45 and 50 (One hundred percent of families in common). Within a common family, three levels of common clones were also simulated: 2, 6 or 10. Half of the 10 ramets of a common clone were planted in environment 1 and half of the ramets planted in environment 2. Clones that were not in a common family were only planted either at environment 1 or environment 2. The experiment layout was a randomised complete block design. A trait was simulated with a narrow-sense heritability of 0.4 and a genetic correlation of 0.7 among the two environments. The simulation was run for 1,500 replicates. The average genetic correlation between environments reduced to 0.58 among 500 clones of 50 families across all replicates.

Phenotype (y) was simulated with a simplified genetic model as the sum of the additive genetic effects (*a*), the residual effects (*ε*) and population mean (*μ*) as Eq (2). No dominance effects and epistatic effects were simulated. The variance of the trait between different ramets of the same clone was equal to zero as well.

y=μ+a+ε (2)

where *a* are sampled from a multivariate normal distribution with ***a***~*N*(0,***G*_0_**), where $G_{0}=\left[\begin{array}{cc} \sigma_{A_{1}}^{2} & \sigma_{A_{1}A_{2}}\\ \sigma_{A_{1}A_{2}} & \sigma_{A_{2}}^{2} \end{array}\right]=\left[\begin{array}{cc} 100 & 99\\ 99 & 200 \end{array}\right]$, where $\sigma_{A_{1}}^{2}$ is the additive genetic variance for environment 1, $\sigma_{A_{2}}^{2}$ is the additive genetic variance for environment 2, $\sigma_{A_{1}A_{2}}$ is the additive genetic covariance between environment 1 and environment 2; *ε* are sampled from a multivariate normal distribution with *ε*~*N*(0,*R*_0_⊗***I***), where $R_{0}=\left[\begin{array}{cc} \sigma_{\varepsilon_{1}}^{2} & 0\\ 0 & \sigma_{\varepsilon_{2}}^{2} \end{array}\right]=\left[\begin{array}{cc} 250 & 0\\ 0 & 300 \end{array},\right]$, where $\sigma_{\varepsilon_{1}}^{2}$ is the residual variance for environment 1. $\sigma_{\varepsilon_{2}}^{2}$ is the residual variance for environment 2; and the population means for two environments are equal to zero with $\mu =\left[\begin{array}{c} 0\\ 0 \end{array}\right]$.

Additive genetic correlation between two simulated environments was estimated using the unstructured model in ASReml-R [40] and calculated as Eq (2). Linear mixed model used to estimate the additive genetic correlation was as Eq (3):

$$y=Xb+Z_{a}u+e$$ (3)

where the additive genetic effects ***u*** have a normal distribution with ***u***~*N*(0,***G*_*A*_**⊗***A***), where $G_{A}=\left[\begin{array}{cc} \sigma_{a_{1}}^{2} & \sigma_{a_{1}a_{2}}\\ \sigma_{a_{1}a_{2}} & \sigma_{a_{2}}^{2} \end{array}\right]$, where $\sigma_{a_{1}}^{2}$ is the additive genetic variance for environment 1, $\sigma_{a_{2}}^{2}$ is the additive genetic variance for environment 2, $\sigma_{a_{1}a_{2}}$ is the additive genetic covariance between environment 1 and environment 2; the residual effects ***e*** have a normal distribution with ***e***~*N*(0,*R*⊗***I***), where $R=\left[\begin{array}{cc} \sigma_{e_{1}}^{2} & 0\\ 0 & \sigma_{e_{2}}^{2} \end{array}\right]$, where $\sigma_{e_{1}}^{2}$ is the residual variance for environment 1. $\sigma_{e_{2}}^{2}$ is the residual variance for environment 2. Genetic correlations between two environments (*r*_*g*_) were estimated as Eq (4) [42]: :

$$r_{g}=\frac{\sigma_{a_{1}a_{2}}}{\sqrt{\sigma_{a_{1}}^{2}\sigma_{a_{2}}^{2}}}$$ (4)

The standard error of estimated genetic correlation was obtained from ASReml-R outputs. The deviation of the additive genetic correlation between environments from zero was tested using a two-tailed likelihood ratio test, by comparing Log Likelihood from models of either adding or dropping the term for the covariance between environments ($\sigma_{a_{1}a_{2}}$).

## Results

### Statistical description of phenotypes

DBH and height showed a growth pattern of having higher phenotypic values and larger variances with increasing age (Table 1). The overall average DBH was 1.67 cm at 28 months of age and 24.02 cm at age 99 months. The average total height varied from below 1.65 m at 26 months of age to 14.92 m at age 99 months. The percentage of needles affected by *Dothistroma* did not show a consistent pattern of change with an increase in age. The overall average percentage of needles that were not affected by *Dothistroma* was about 60–90% during the period 28–65 months of age.

### Connectivity between trials

**Fig 1:**
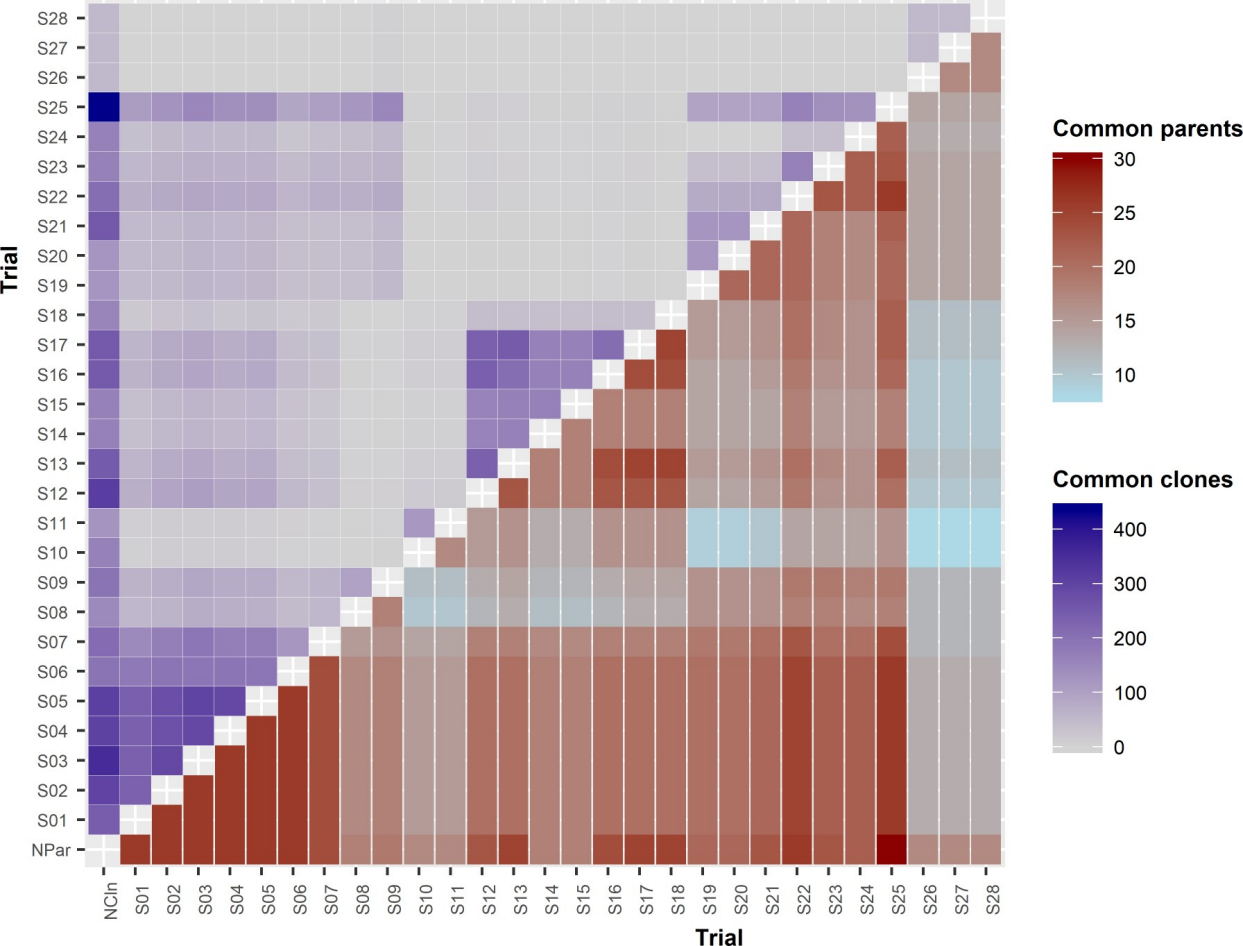
Number of common parents (below diagonal) and number of common clones (above diagonal) between trials. Number of parents within each trial is shown on the bottom row with ‘NPar’ as trial name and number of clones with each trial is shown on the first column from the left with ‘NCln’ as trial name.

The number of parents in these trials ranged from 17 to 30 with an average of 21 (Table 1 and Fig 1). The percentage of parents in common between all possible pairs of trials ranged from 47% to 100% with an average of 79%. In the NZ 2005 trial series (S26, S27 and S28), there were 17 parents altogether, 13 parents for the full-sib progeny and 4 parents for the clones. The average number of clones in all trials was 213 with a range of 54 to 420. The average percentage of clones in common between all possible pairs of trials was 22% with a range of 0 to 47%. There was good connectivity among trials which were established in the same year. The trials established in New Zealand during 1999 and 2000 (S10-S18) had poor connectivity with other trials. The trials established in New Zealand in 2005 (S26, S27 and S28) had no common clones tested in other trials. The best connectivity was between trials S01-S07.

### Estimates of heritability

The narrow-sense and broad sense heritabilities estimated for these traits were quite different from trial to trial. The narrow-sense heritability for DBH ranged from 0.01 to 0.31 with an average of 0.11. Small additive genetic variances were estimated in four trials. Considerable non-additive genetic variation was estimated in DBH which mostly led to moderate-to-high estimates of broad-sense heritability. The average broad-sense heritability for DBH was 0.27 with a range of 0.01 to 0.45. A similar pattern was observed for the narrow-sense and broad-sense heritabilities for height. There were small additive genetic variances for height in two trials. The average narrow-sense heritability for height was 0.21 with a range of 0.01 to 0.46 and the average broad-sense heritability was 0.34 with a range of 0.11 to 0.57. Five trials were measured for *Dothistroma* resistance. Heritabilities for *Dothistroma* resistance were estimated in these trials, all of which were in New Zealand. Narrow-sense heritability was 0.30 on average with a range of 0.16 to 0.49, and broad-sense heritability was 0.40 with a range of 0.24 to 0.49.

### Genotype by environment interaction

**Fig 2:**
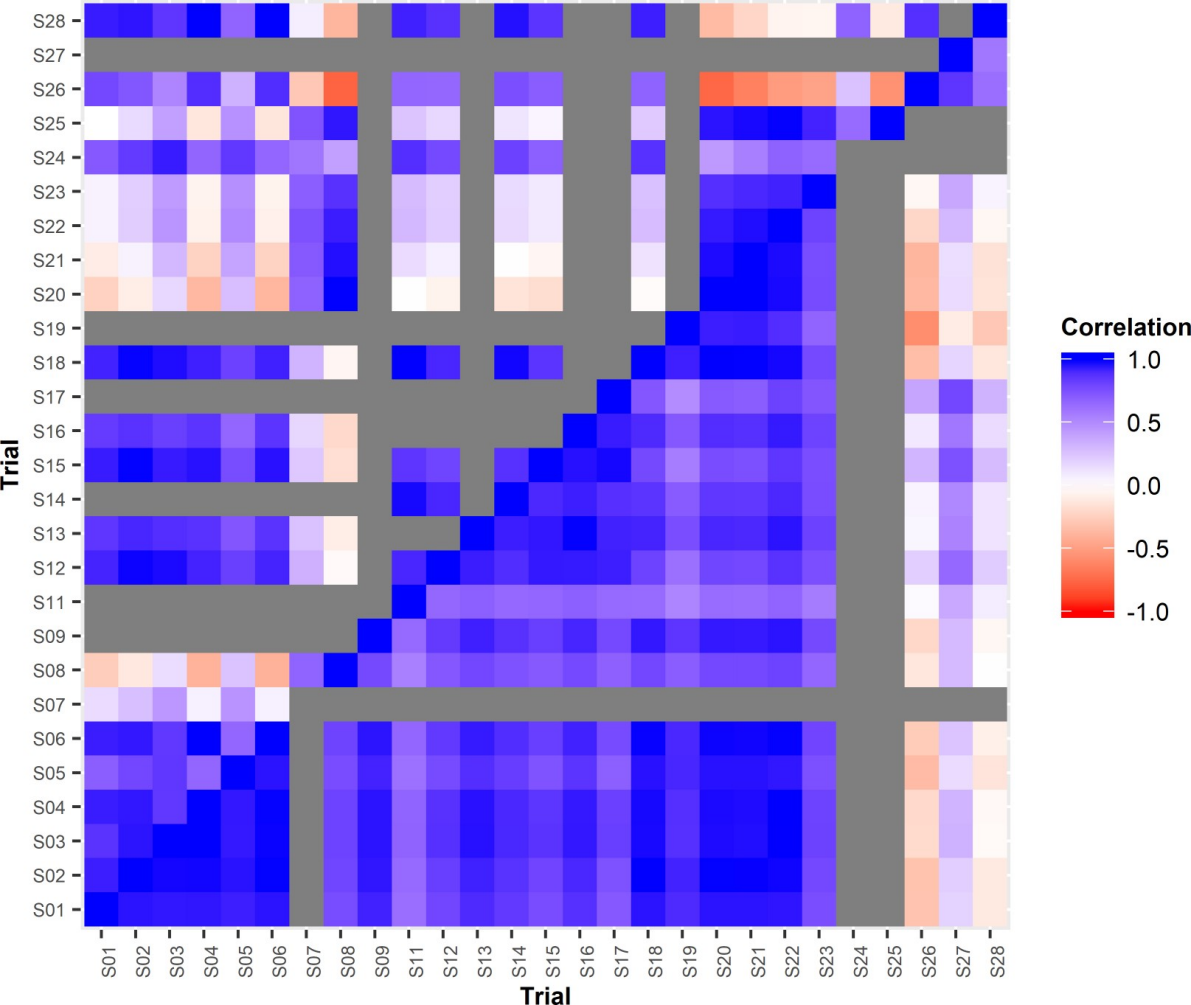
Heat-map of the additive genetic correlation matrices between trials for DBH (above diagonal) and for height (below diagonal), estimated from the FA2 model. Level of G×E between trials is measured by the genetic correlation. The higher the genetic correlation the lower is the level of G×E between trials. Dark grey indicates genetic correlation was not estimated.

The FA model with an order k = 2 explained 93.73%, 88.02% and 88.33% of the variances for DBH, height and DO_R, respectively. The heatmaps in Fig 2 show the additive genetic correlations between trials for DBH and height and the heatmaps in S1 Fig show the additive genetic correlations between trials for *Dothistroma* resistance that were obtained from the FA2 model. Apparent high G×E interactions were observed between some trials for DBH. The average genetic correlation for DBH between pairwise trials was 0.40 with 30% of pairs over 0.7, 27% in a range of 0.4–0.7 and 43% below 0.4. Apparent G×E interaction level for height was also high between some trials, but not as high as for DBH. The average genetic correlation for height between trials was 0.61, with 55% of pairwise genetic correlations over 0.7, 22% in a range of 0.4–0.7 and 23% below 0.4. High levels of apparent G×E were also found for *Dothistroma* resistance between some trials. The average genetic correlation between trials for *Dothistroma* resistance was 0.44 with 40% pairwise genetic correlations over 0.70, 30% at a range of 0.4–0.7 and 30% below 0.40.

There were patterns of genetic correlations between trials. For DBH, higher genetic correlations were observed within trial series planted in the same year, e.g. the NZ 2001 trial series (S20–S24) (Fig 2). For height, low genetic correlations were observed between the NZ 2005 trial series (S26-S28) and other trials (Fig 2). For *Dothistroma* resistance, high genetic correlations were observed among NZ trials S14, S19 and S20 and low genetic correlations between trials S10 and S25 and trials S14, S19 and S20 (S1 Fig).

**Fig 3:**
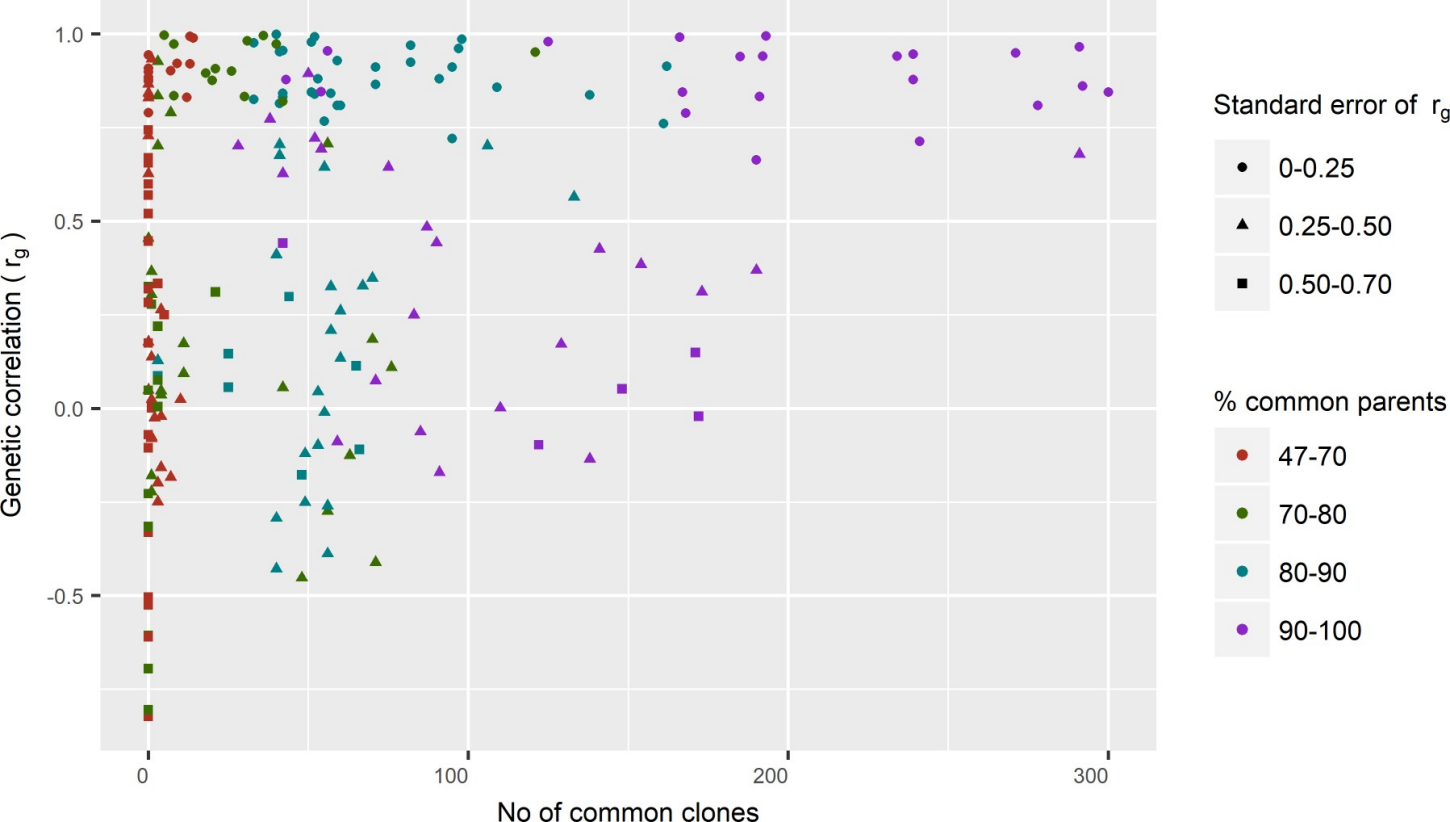
Relationship between additive genetic correlation estimated using the FA2 model and connectivity between trials through common parents and common clones for DBH.

When the percentage of common clones tested between trials was above 25%, genetic correlation between these trials ranged from -0.08 to 1.00 (Fig 3). When the percentage of common clones tested between trials was 30% or lower, a higher number of negative genetic correlations between trials was observed. Even more negative genetic correlations were observed when the percentage of common clones fell below 10%. These results indicate that the connectivity between trials played a big role in the estimation of G×E interactions, and that the estimated low genetic correlations between some trials might also be due to low connectivity. The results also suggested that the estimates of genetic correlations between trials might not be reliable when the connectivity between trials is low. For the trials between which there were at least 30% clones in common, the average genetic correlation was 0.63 (-0.08–0.98) for DBH, 0.63 (0.30–1.00) for height and 0.73 (0.60–0.90) for DO_R. Thirty percent or more clones in common appears to provide sufficient connectivity between trials and leads to moderate levels of estimated G×E interaction for growth traits between trials.

Standard errors of genetic correlations between trials tended to be higher when there was a lower number of clones or parents in common (Fig 3). When the number of common clones between trials was less than 200, standard errors of some genetic correlation estimates were larger than 0.25, even when the number of parents in common was above 90%. When the number of common clones was less than 100, more than half of the pair-wise genetic correlations between trials had very high standard errors (0.25–0.70). It appears that low estimates of between-environment genetic correlation with high standard errors were related to low connectivity between environments.

### Simulation results

**Fig 4:**
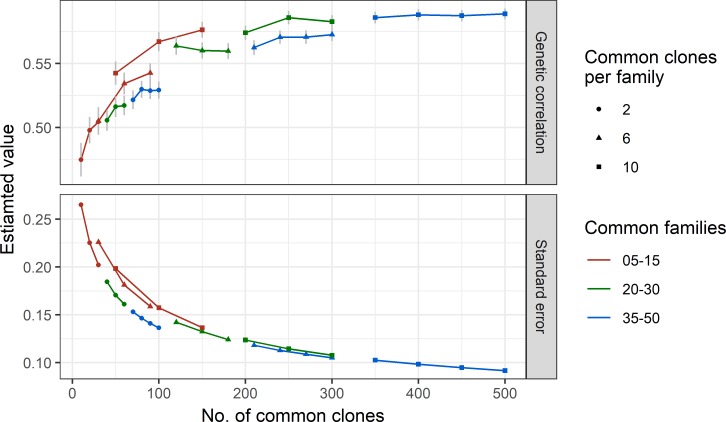
Relationship of estimated genetic correlation and its standard error with the connectivity between environments, through the common families (5–15, 20–30 or 35–50) and the common clones (4, 6 or 10 per family), in the simulated data.

Estimates of genetic correlation between environments and their standard errors were shown in Fig 4 for different levels of connectivity through common clones and common families. When 35–50 families were in common and with 10 common clones per family, the estimate of between-environment genetic correlation was about 0.58 and its standard error estimate was below 0.1. In this case, genetic correlation was able to accurately estimated. With the decreases of connectivity through common families and common clones, the estimates of between-environment genetic correlation reduced and the standard error estimates of the correlations increased dramatically. When the number of common families was 5–15 with 2 common clones per family, the genetic correlation estimate decreased to around 0.5 and standard error estimate of the correlation was increased to 0.2–0.27. This meant that for the same true level of G×E between environments, a low level of G×E was observed when the number of common clones between environments was above 300 and a high level of G×E was observed when the number of common clones between environments was below 100.

Table 2 shows the percentage of cases in the simulation where the estimates of genetic correlation between environments were significantly different from zero. When the number of common families was 5–15, there were 47–76% cases where genetic correlation estimates between environments were significantly different from zero. When the number of common families was 30 or less and the number of common clones was 4, there were 47–80% of cases where genetic correlation estimates were significantly different from zero. When the number of common families was 35–50, there were 92–100% cases where genetic correlation estimates were significantly different from zero.

**Table 2:** Percentage of estimates of genetic correlation between environments that were significantly different from zero.

No. of common families	No. of common clones/family		
	4	6	10
05–15	47%	57%	76%
20–30	80%	92%	99%
35–50	93%	100%	100%

## Discussion and conclusions

This study has shown that the precise estimation of genetic correlations between trials depends on the levels of connectivity between trials. Apiolaza [34] found an association between the number of parents in common across trials and the magnitude of the standard error of the estimated genetic correlation, and that the estimated correlation tended to be under-estimated when there were fewer parents in common. In the current study with clonal trials, the number of parents was 17–30 with an average of 21. The percentage of parents in common was 47–100% with an average of 78%. The connectivity between trials via parents was, therefore at a reasonable level. However, it should be noted that the trials in this study were not designed to screen parents but rather to screen the individual clones. Despite this complication, the current study clearly showed that there was a positive association between the number of clones in common across trials and the size of the estimates of genetic correlations between trials. Fig 3 showed a trend for the estimated correlation tending to be lower when the percentage of clones in common between trials was low, which means that the genetic correlations might be not reliable in these cases. Apiolaza (28) also reported that any pair of trials with less than 20 parents in common (either directly or via previous generations in the pedigree) caused convergence problems in obtaining estimates of genetic correlation. The current study also expressed convergence problems in estimating pairwise genetic correlations when the number of clones in common was low.

The simulation results clearly showed the same pattern, that is, that the lower the connectivity between environments the lower the estimate of genetic correlation and the higher the estimate of its standard error. In the simulation, a low level of G×E between two environments was simulated but the estimated genetic correlation showed a high level of G×E when the number of common clones between environments was below 100. These results suggest that the number of genetic entities in common between trials is a key factor in future experimental designs for estimating G×E interaction. Low connectivity via common genetic entities between trials might lead to underestimated genetic correlations and, therefore, an over-estimated level of G×E interaction. In other words, these results indicate that if there is insufficient connectivity between trials, they should not be used in to identify the level of G×E in radiata pine.

The simulation results showed that a lower connectivity between environments led to a lower estimate of genetic correlation with a higher standard error, compared to the situation with a higher connectivity between trials in different environments. Moreover, there was a high percentage of cases where the estimate of genetic correlation was not significantly different from zero. If a genetic correlation estimate was not significantly different from zero, it can mean that genetic correlation between environments could not be reliably estimated with the given sample size and connectivity between environments. A trial designed with 50 families planted in two environments and used to estimate the level of G×E between the environments may be considered acceptable, since there is over a 90% chance that the genetic correlation between environments can be estimated. Therefore it is recommended that, infuture, for detection of the level of G×E for a trait in a trial it should have at least 50 families and 10 clones per family, where at least 6 clones per family are in common between environments when 20–30 families are in common and at least 4 clones per family are in common when 35 or more families are in common. This is equivalent to at least 30% of clones needing to be in common between environments, which was a similar threshold of connectivity found in Fig 3. We could also conclude that any two trials used in this study were not suitable to be used for estimating G×E where the number of common clones between them was less than 30%.

G×E interactions are generated when differences exist between environments [21]. It has been suggested that the larger the difference between environments, the higher the G×E interaction might be between the environments [43], although this must be tested for each specific species, trait, and environment combination. Identification of environmental variables that either contribute to, or are related to the creation of G×E interaction in forest trees has been studied in the literature. These variables included climatic and geographic variables, such as temperature [27,34,44], rainfall [37], longitude, latitude and elevation [25]. Differences in soil properties, such as soil type [45,46] and soil nutrient content [39], can also result in G×E interactions. There are huge numbers of combinations of different environmental variables that could generate G×E interactions for given genotypes, or for any genotypes.

Forest tree breeders are interested in having information of G×E patterns in the forest in order to make decisions on where to deploy genotypes to maximize genetic gain. There are two ways that tree breeders can obtain information to reveal the patterns for breeding and deployment. One easy way of identifying G×E interaction in tree breeding is to screen the performance of a set of genotypes across a set of commercially important environments. In this way, breeders can identify which genotypes are not sensitive to the environments, or alternatively, which genotypes are the best match to each of the environments. The second way is more complicated and requires long-term efforts to achieve the goal of increased overall genetic gains. It would require all of the unique environments within forest estates in a country or a region to be identified and G×E interaction patterns to be fully examined in trials andunderstood for any genotypes in a breeding population. The G×E patterns can be caused by either of, or a combination of, rank-change interactions or variance differences between these unique environments [20]. Identifying such G×E interaction patterns for a given forest is likely to be a huge task to complete. G×E studies instead usually involve use of a limited number of sites to represent the whole plantation. In the current study, we tested 28 sites in forests in New Zealand and Australia. However, the G×E patterns identified in the current study only reflect G×E for the sites tested and might not reflect the patterns of G×E in all the sites in theplantations in which we are interested. Extending this line of thinking seems to suggest that we may need many more sites to be tested in the radiata pine forest plantations in New Zealand and Australia in order to accurately identify and capture the gain available from a knowledge of G×E interactions. It is worth exploring the validity of this conclusion further by examining the magnitude and underlying causes of G×E interactions in traits of interest in radiata pine.

Genetic variance in growth traits, expecially DBH, has been studied extensively in New Zealand and Australia. The average narrow-sense heritability for DBH varied from 0.03 to 0.88 among 38 studies, with a grand mean of 0.23 for all studies while the average estimate of broad-sense heritability was 0.39, or almost twice the average estimated narrow-sense heritability [47]. Clonal repeatability estimates ranged from 0.05 to 0.33 for DBH and from 0.09 to 0.31 for height [24]. The broad-sense heritability ranged from 0.01 to 0.45 with an average of 0.27 in this study. Narrow-sense heritability for DBH was 0.06–0.27 in two trial series planted at four sites in New Zealand [48,49].

G×E for growth traits has been extensively studied in New Zealand and Australia. Family × site interaction variance components for stem volume were highly significant, statistically and biologically, between two pumice soil sites and two phosphate-retentive clay soil sites [32]. The correlations of parental breeding value estimates for DBH between 11 widely diverse sites across New Zealand was 0.41 (-0.22–0.74) in a progeny test [28]. The ratio of estimated interaction to genetic variance for DBH was 0.70 in a progeny test with 25 parents planted across 11 widely diverse sites across New Zealand [28], 1.51 in a progeny test of 170 open-pollinated families from second-generation plus trees planted in four sites in New Zealand [32], 2.0 in an open-pollinated progeny test planted in 10 sites across Australia [50] and above 0.50 in a diallel experiment covering 10 sites in Australia [23]. The genetic correlation estimate between pairs of environments for DBH was 0.39 [23], 0.34–0.38 [51] and -0.60–1.0 [25]. Baltunis and Brawner [24] reported high G×E for growth traits in clonal trials among Australian sites but not among New Zealand sites. Nearly two-thirds of genetic correlation estimates for DBH between paired sites were below 0.6 in an analysis using data covering 76 sites across the whole of New Zealand [27]. Cullis, Jefferson [26] suggested the existence of substantial additive G×E for DBH using factor analytic models.

Significant genetic variation in *Dothistroma* resistance has been identified in a number of studies and was also apparent in the current study. The narrow-sense heritabilities reported in the current study were in the range reported in the published literature. Wilcox [52] reported a narrow-sense heritability for *Dothistroma* resistance of 0.30 with strong additive genetic variance, indicating that straightforward selection of resistant trees would give predictable improved resistance in the offspring. Carson [53] reported an average narrow-sense heritability for *Dothistroma* resistance of 0.24 across four sites. A narrow-sense heritability estimate of 0.18 for *Dothistroma* resistance in radiata pine was estimated for a factorial cross which included six full-sib families [54]. In a series of 16 radiata pine trials assessed in Australia the estimated narrow-sense heritability ranged from 0.05 to 0.69 with a median of 0.36 [16]. Kennedy and Yanchuk [49] reported a heritability of 0.20–0.34 for *Dothistroma* resistance in radiata pine. These estimates demonstrate that genetic gain can be achieved with selection on additive genetic variance.

In the literature, G×E interactions for disease resistance have been reported in radiata pine and other species. An apparent level of G×E interaction was estimated for *Dothistroma* resistance in the current study, but after considering the lack of connectivity between trials it is likely that the level of interaction is much less important than the genetic parameter estimates suggest. This is consistent with what is reported in the literature for G×E interaction in *Dothistroma* resistance in radiata pine. Shelbourne [55] suggested that interactions will have a serious effect on gains from selection and testing when the interaction variance reaches 50% or more of the genetic variance. Carson [53] reported that there was little evidence for seedlot × environment interaction in *Dothistroma* resistance, indicating that seedlot rankings obtained on one site will be reliable for overall ranking of parents in radiata pine. The ratio of additive genetic variance × site interaction variance to additive genetic variance for *Dothistroma* resistance was found to be 0.57 in radiata pine [28]. This suggested that exploiting G×E to achieve gain in *Dothistroma* resistance may not be the best strategy. In contrast, high levels of G×E interactions have been reported for other diseases. A significant G×E interaction for needle retention in radiata pine was observed with an interaction variance to additive variance ratio of 0.86 [28]. In loblolly pine (*Pinus taeda*), Li and Mckeand [56] reported highly significant G×E for percent fusiform rust infection (*Cronartium quercuum* (Ber.) Miyabe ex. Shirai f. sp. *Fusiforme*) at 21 test locations in coastal Georgia, Florida, Alabama, and Mississippi, but only a few interacting families had regression coefficients significantly different from the expected value of 1. A low level of G×E for rust incidence was reported for loblolly pine planted in the Lower Coastal Plain of the Southeastern USA [57]. In slash pine (*Pinus elliotti*), genetic correlations between sites were over 0.67 for fusiform rust resistance and G×E did not appear to be important in rust resistance in the United States [58]. It is interesting to note that extensive genetic variation was observed among fusiform rust isolates and in the response of loblolly pine to these isolates [59], whereas in contrast there was no genetic variation at all observed among collections of *Dothistroma pini* in New Zealand in a genetic diversity study [60]. This is consistent with a hypothesis that genetic variation in the pathogen could contribute to higher levels of G×E in resistance of the host population [61].

One limitation of this study was that the assessment age of DBH, height and *Dothistroma* resistance of genetic material ranged from 28 months to 99 months. Ideally, the age of performance assessment across all trials should be at the same age. In forest tree breeding, resources required for establishing trials are always limited. Each year only a small number of trials are able to be established. Genetic analyses usually use trials established during a number of years or even all trials that were ever established [26]. The assessment age of performance also varied. For example, the genetic material used in this paper was established during a period between 1999 and 2005, and was deployed in Australia and New Zealand. Assessment of performance at an early age makes it possible to conduct selection at an early age. The benefit of early selection is to reduce generation interval and increase genetic gain per unit of time [21]. From one example in the literature, genetic correlation for 30 open-pollinated families tested at two sites between early age (age 3 after planting) and age 26 reached 0.8 for growth traits [62], suggesting that analysingssessments of performance at multiple ages did not create significant G×E or did not create bias for estimating site-site genetic correlation.

The model used in the simulation was simplified to show the relationship between connectivity and the estimates of additive genetic correlation between environments. We found that lower connectivity between environments led to a lower estimate of additive genetic correlation with a higher estimate of standard error of the genetic correlation. Variances for dominance effects, epistatic effects and variance between ramets of the same clones were not considered in the simulation. The relationship between connectivity and the estimation of additive genetic correlation would likely be unchanged if these terms were included, but model complication would increase. An attempt was made to include the dominance effects in the simulation of phenotypes and in the estimation model. However, ASReml-R was sometimes not able to converge, which might suggest that the simulated population size was not large enough to estimate dominance effects. A large simulated population size may be needed to estimate dominance effects and an even larger population size may be needed if epistatic effects are included. In addition, in the future, simulations with various population sizes tested at more than two environments may be needed to investigate how many clones need to be in common between environments for obtaining reliable estimates of genetic correlation and G×E.

### Implications for radiata pine breeding

G×E interaction appears to exist for growth traits in radiata pine genetic trials. However, this study suggests that the magnitude of G×E interactions found in radiata pine clonal trials was often overstated due to a number of factors, including particularly the lack of connectivity between environments, especially lack of connectivity with common genetic entities tested across multiple environments. The implication of the results for growth rate in the current study for radiata pine breeding in New Zealand and Australia is that a strategy does need to be developed to deal with G×E, either by selecting stable genotypes that show high performance across multiple environments or by selecting the best genotypes for each environment to maximize genetic gain in each specific environment. Given the substantially increased resources that would be required to achieve the latter, it is likely that the former is the best strategy, that is, selecting for the best set of stable genotypes. This is because the requisite increase in resources for estimating gain in specific environments to capture G×E could alternatively be used to increase gain from selecting stable genotypes more effectively. The results in the current study also suggest that connectivity between trials with common genetic entities that are tested across environments needs to be considered when designing trials and maximizing gain. When designing clonal trials with a population size similar to the trials used in this study in multiple environments to accurately estimate the level of G×E, and where resources for creating connectivity between environments are limited, at least 30% of clones need to be in common between environments.

## Supporting information

S1 Fig Heat-map of the additive genetic correlation matrices between trials for DO_R (above diagonal), estimated from the FA2 model. Level of G×E between trials is measured by the genetic correlation. The higher is the level of genetic correlation the lower the level of G×E between trials.
(TIFF)
