# Supplementary figures and images for: Genotype by environment interaction for growth and Dothistroma resistance and clonal connectivity between environments in radiata pine in New Zealand and Australia
Source: PLoS One. 2018 Oct 12;13(10):e0205402. doi: 10.1371/journal.pone.0205402 (PMC6185738; doi:10.1371/journal.pone.0205402)

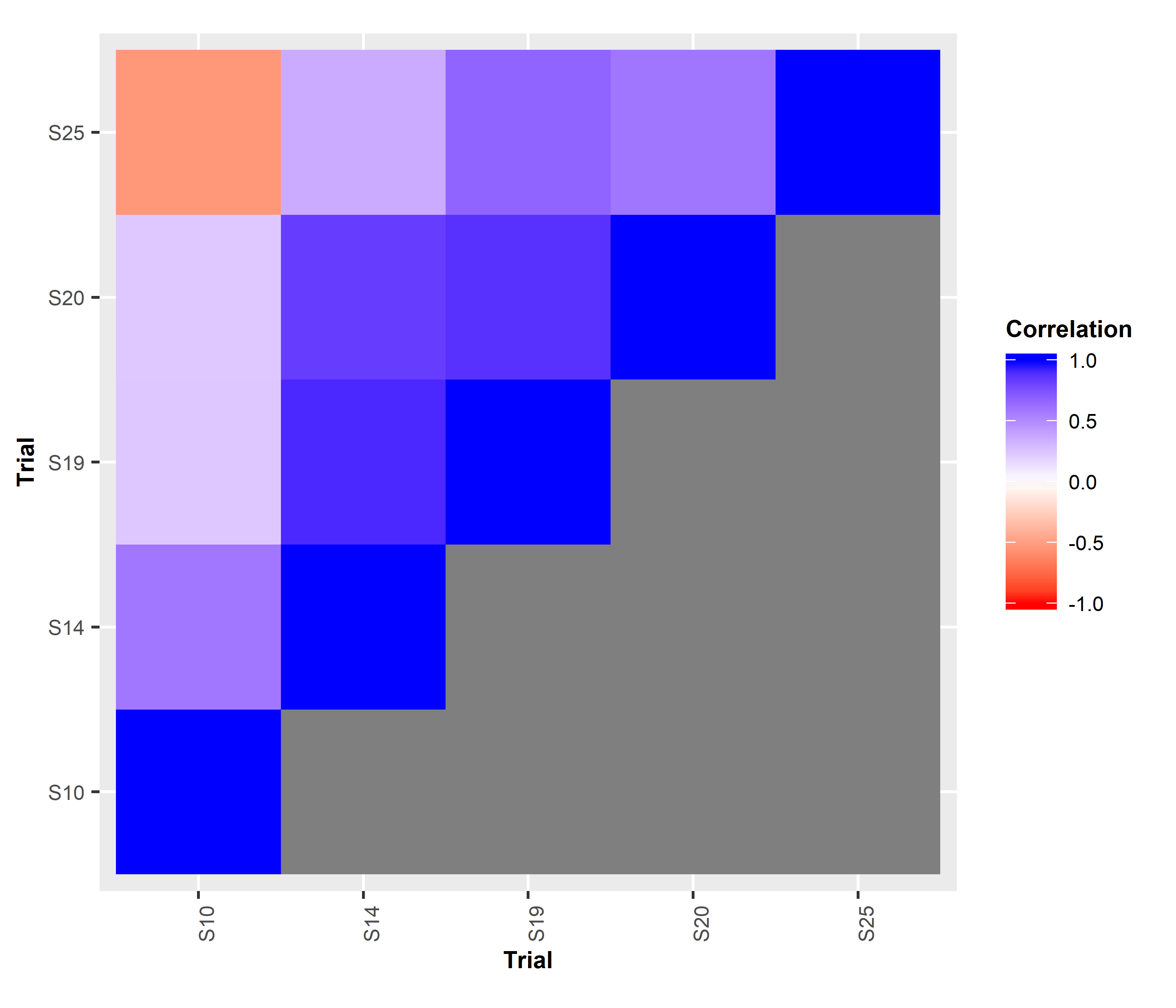

Supplement: S1 Fig — Level of G×E between trials is measured by the genetic correlation. The higher is the level of genetic correlation the lower the level of G×E between trials. (TIFF) [file pone.0205402.s001.tiff]
